# Overexpression of WAX INDUCER1/SHINE1 Gene Enhances Wax Accumulation under Osmotic Stress and Oil Synthesis in *Brassica napus*

**DOI:** 10.3390/ijms20184435

**Published:** 2019-09-09

**Authors:** Ning Liu, Jie Chen, Tiehu Wang, Qing Li, Pengpeng Cui, Chengxi Jia, Yueyun Hong

**Affiliations:** National Key Laboratory of Crop Genetic Improvement, Huazhong Agricultural University, Wuhan 430070, China (N.L.) (J.C.) (T.W.) (Q.L.) (P.C.) (C.J.)

**Keywords:** WAX INDUCER1/SHINE1 (WIN1), wax accumulation, salt stress, oil synthesis, transcriptional regulation, *Brassica napus*

## Abstract

WAX INDUCER1/SHINE1 (WIN1) belongs to the AP2/EREBP transcription factor family and plays an important role in wax and cutin accumulation in plants. Here we show that BnWIN1 from *Brassica napus* (Bn) has dual functions in wax accumulation and oil synthesis. Overexpression (OE) of *BnWIN1* led to enhanced wax accumulation and promoted growth without adverse effects on oil synthesis under salt stress conditions. Lipid profiling revealed that *BnWIN1*-OE plants accumulated more waxes with elevated C29-alkanes, C31-alkanes, C28-alcohol, and C29-alcohol relative to wild type (WT) under salt stress. Moreover, overexpression of *BnWIN1* also increased seed oil content under normal growth conditions. BnWIN1 directly bound to the promoter region of genes encoding biotin carboxyl carrier protein 1 (BCCP1), glycerol-3-phosphate acyltransferase 9 (GPAT9), lysophosphatidic acid acyltransferase 5 (LPAT5), and diacylglycerol acyltransferase 2 (DGAT2) involved in the lipid anabolic process. Overexpression of *BnWIN1* resulted in upregulated expression of numerous genes involved in de novo fatty acid synthesis, wax accumulation, and oil production. The results suggest that BnWIN1 is a transcriptional activator to regulate the biosynthesis of both extracellular and intracellular lipids.

## 1. Introduction 

*Brassica napus* (*B. napus*) is the second largest oilseed crop widely grown in the world behind only soybean. It provides high-quality edible oil for human health, renewable industrial feedstock, and bioenergy [1,2]. The production of *B. napus* suffers frequently environmental stresses during growth seasons. High salinity, featured with increased sodium concentration in cells, is one of the major adverse factors inhibiting plant growth and development [3]. Plants are sessile and have evolved to cope with different stresses via diverse changes including morphological modification and physiological, biochemical, or molecular responses [4]. One of such changes is wax accumulation in terrestrial plants in response to osmotic stress [5,6]. Waxes, located either outside of the cuticle or within the cuticular matrix, function as a hydrophobic barrier in preventing water loss from non-stomatal transpiration in plants and are important for plant tolerance to drought [7,8,9]. Waxes also have sophisticated roles in the protection against pathogen infection, insect attack, and ultraviolet radiation [10,11]. In addition, epicuticular waxes prevent organs from fusion and restrict the deposition of dust and other air pollutants [5,6,12].

Epicuticular waxes are mostly composed of very-long-chain fatty acids (VLCFAs), alkanes, aldehydes, alcohols, ketones, and esters, which are derived from fatty acids [13,14,15]. Fatty acids are synthesized by FA synthase (FAS) complex in plastids [16]. The acetyl-CoA carboxylase (ACCase) complex, consisting of biotin carboxyl carrier protein (BCCP), biotin carboxylase (BC), and carboxyltransferase (CT), uses acetyl-CoA and CO_2_ to produce malonyl-CoA, the first committed step in de novo FA synthesis [16,17]. After being released from FAS and exported from plastids, free FAs are activated to acyl-coenzyme A (CoA) by long-chain acyl-CoA synthetases (LACSs) for further elongation by the fatty acyl-coA elongase (FAE) complex in the endoplasmic reticulum (ER) to produce VLCFAs, with chain lengths ranging from 20 to 34 carbons [18]. VLCFA derivatives, the major constituents of waxes, are synthesized via two independent pathways using VLCFAs as substrates in ER: the alcohol pathway that produces very-long-chain VLC-fatty primary alcohols and their esters, and the alkane-forming pathway by decarbonylation to generate VLC-alkanes, secondary n-alcohols and ketones [13,14,19,20]. Wax constituents synthesized in the ER are transported to the plasma membrane, exported to the epidermal cell wall, and ultimately loaded in the outer surface of aerial plant tissues [21].

Wax content and composition differ depending on plant species, tissues, environmental stresses, and development stages, which are modulated by multiple metabolic processes [14,22]. Numerous enzymes responsible for wax synthesis have been identified through mutant screening, and mutations in these genes exhibited reduced wax accumulation [7,8,9,23,24,25]. For example, ECERIFERUM3 (CER3) catalyzes the reduction of VLCFA to produce fatty aldehydes, whereas CER1 catalyzes the decarbonylation of fatty aldehydes to *n-*alkanes. Loss of function in the CER1 and CER3 leads to a substantial decrease in alkanes [25,26]. CER2 plays a role in fatty acid elongation with chain length from C28 to C34 [27,28]. LACS1 and LACS2 are responsible for activation of VLC fatty acids for wax components accumulation. The cuticular wax in Arabidopsis *lacs1* and *lacs2* mutants was decreased [29]. Diacylgycerol acyltransferase 2 (DGAT2) catalyzes the ester formation between fatty acids and alcohols, and loss of DGAT2 leads to reduced wax accumulation [19]. Arabidopsis CER5 is responsible for wax transport, and mutation in *CER5* leads to reduced epicuticular wax and increased intracellular wax content [30,31]. These results suggest that wax accumulation is modulated by diverse regulatory processes.

Due to the complicated networks of wax anabolism, transcription factors may play important roles through regulating coordinately the induction of multiple metabolic pathways [7,32]. WAX INDUCER1/SHINE1 (WIN1/SHN1), containing AP2/ERF domain binding to the GCC (AGCCGCC) box and dehydration responsive element (DRE) of target genes, was first identified to be important for wax accumulation and was involved in drought tolerance in *Arabidopsis* [7,8]. Subsequently, WIN1-related members in the AP2/ERF family, such as rice OsWR1 and Medicago WXP1, were found to play a positive role in wax accumulation and drought tolerance [33,34]. Arabidopsis WRINKLED4 (WRI4), another AP2/ERF transcription factor, also increases the expression of genes involved in wax accumulation [35]. These results suggest that WIN1 and its related transcription factors are important for extracellular lipids synthesis. However, the role of WIN1 in intracellular lipids remains to be elucidated. Here, we show that BnWIN1 from *B. napus* has effects on both wax and storage lipid accumulation. Overexpression (OE) of *BnWIN1* results in enhanced wax accumulation and increased tolerance to salt stress whereas it enhances oil synthesis under normal growth conditions.

## 2. Results

### 2.1. *BnWIN1* Is Expressed in Various Tissues in B. Napus

**Figure 1:**
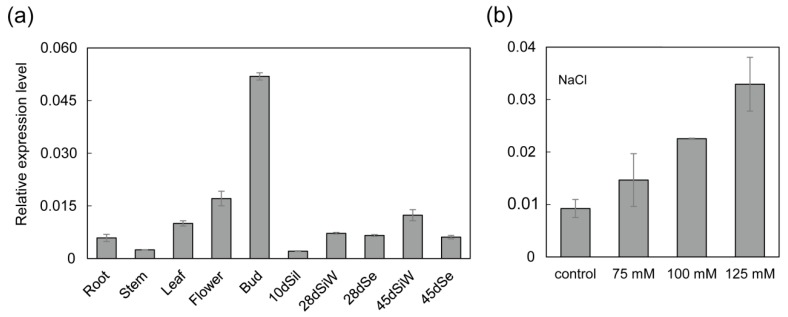
*BnWIN1* is expressed in various tissues and is induced by salt stress in *Brassica napus*. (**a**) The expression pattern of *BnWIN1* in *B. napus* detected by qRT-PCR normalized to *BnActin*. Total RNA was extracted from various tissues of *B. napus*. Values are means ± SD (*n* = 3 separated samples). Bud, flower bud; 10dSil, silique at 10 days after flowering; 28dSiW and 45dSiW, silique wall at 28 and 45 days after flowering, respectively; 28dSe and 45dSe, seed at 28 and 45 days after flowering, respectively. (**b**) The transcript level of *BnWIN1* was induced by NaCl stress in *B. napus.* Total RNA was extracted from 21-day-old plants treated with 0 (control), 75, 100, and 125 mM NaCl for two weeks. *Actin* was used as an internal standard. Values are mean ± SD (*n* = 3 separate samples).

To investigate temporal and spatial distribution of *BnWIN1* mRNA in *B. napus* (Supplementary Figure S1), total RNA was extracted from various tissues of cultivar (cv.) Westar (*B. napus*) plants, and the expression pattern of *BnWIN1* was analyzed by quantitative reverse transcription PCR (qRT-PCR). The *BnWIN1* mRNA was detected in various tissues. The *BnWIN1* transcript level was highest in flower bud relative to other tissues. The *BnWIN1* expression levels in flower, leaf, developing seed, silique wall, and root were higher than in stem and 10-day silique (Figure 1a).

### 2.2. Overexpression of *BnWIN1* Enhanced Salt Stress Tolerance

**Figure 2:**
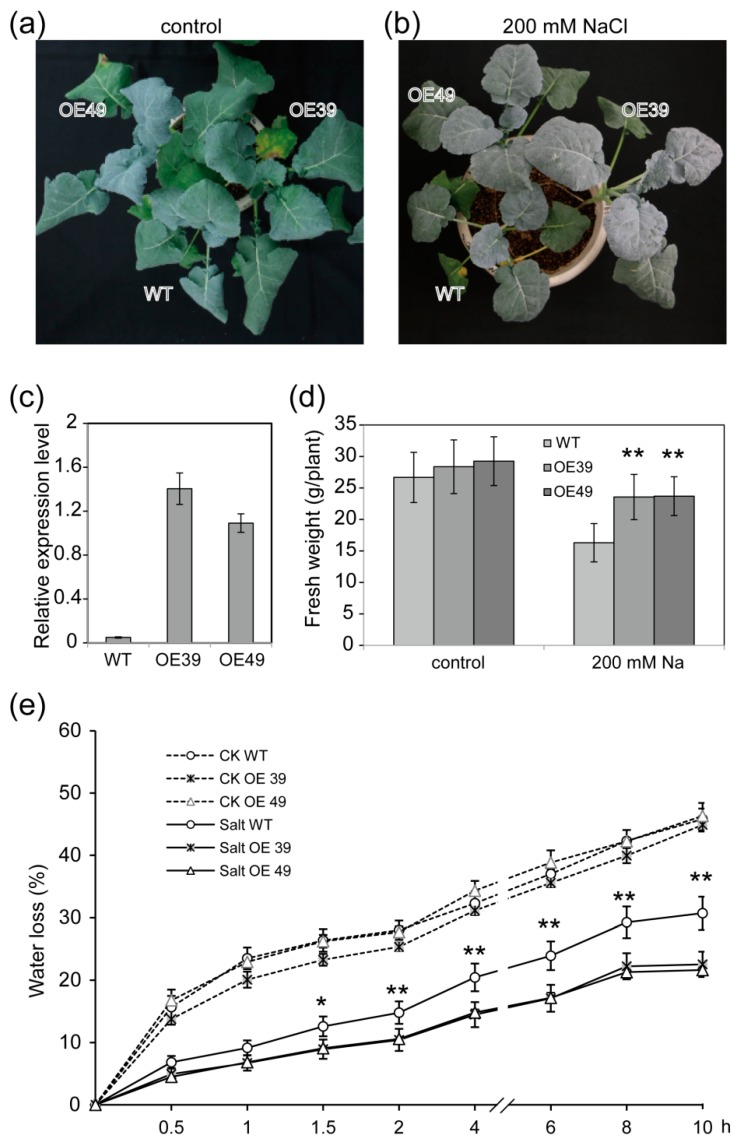
Overexpression of *BnWIN1* enhanced plants growth and reduced water loss under salt stress. (**a**,**b**) The phenotype of *BnWIN1*-overexpression (OE) plants under control and salt stress conditions. Forty-day-old plants grown in soil in pots were treated with 200 mM NaCl for 30 days. (**c**) The *BnWIN1* expression level of *BnWIN1*-OE plants confirmed by real-time PCR with *β-Actin* as an internal standard. Values are means ± SD (*n* = 3 separated samples). (**d**) Enhanced growth of *BnWIN1*-OE plants under salt stress. Forty-day-old plants grown in soil in pots were treated with 200 mM NaCl for 30 days, and fresh weight was measured. Values are means ± SD (*n* = 12 plants). (**e**) Water loss from detached leaves. Values are mean ± SD (*n* = 3 independent experiments). * and ** indicate significant difference at *p < 0.05* and *p < 0.01*, respectively, compared with wild type (WT) plants based on Student’s *t* test.

The *BnWIN1* transcript level was markedly induced by salt stress when plants were subjected to 125 mM NaCl treatment for two weeks, as compared to control conditions without salt stress treatment (Figure 1b). The result suggests that BnWIN1 is involved in salt stress. To test this hypothesis, the full length of *BnWIN1* complementary DNA (cDNA) was cloned from cv. Westar by reverse transcript (polymerase chain reaction) PCR using mRNA extracted from leaves as template and was overexpressed in cv. Westar plants under the control of the 35S promoter. The expression level in *BnWIN1*-OE plants was 21-fold increased as compared to wild type (WT) plants (Figure 2c).

**Figure 3:**
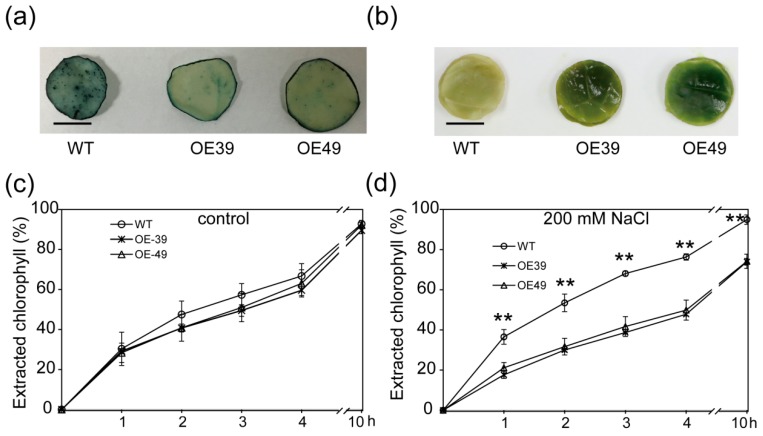
Overexpression of *BnWIN1* reduced cuticular permeability under salt stress. (**a**) The reduced tubulin staining in *BnWIN1*-OE plants under salt stress conditions. Scale bar = 1 cm. (**b**–**d**) The chlorophyll extraction rate of *BnWIN1-*OE and WT plants under control and salt stress conditions. Forty-day-old plants grown in soil in pots were treated without (control) or with 200 mM NaCl for 30 days. Values are mean ± SD (*n* = 3 independent experiments). Scale bar = 1 cm. ** indicate significant difference at *p* < 0.01 compared to WT based on Student’s *t* test.

To investigate whether BnWIN1 is involved in osmotic stress, *BnWIN1*-OE and WT plants were subjected to salt stress. Under control conditions, *BnWIN1*-OE plants were not visibly different from WT plants (Figure 2a,d). However, *BnWIN1*-OE plants grew better than WT when plants were treated with 200 mM NaCl. The fresh weight of *BnWIN1*-OE plants was increased by 45% compared to WT (Figure 2b,d). *BnWIN1*-OE plants exhibited more glaucous appearance in the leaf surface than that of WT plants under salt stress conditions (Figure 2b). Water loss from salt-stressed detached leaves of *BnWIN1*-OE plants was much less than that of WT and, in particular, non-stomatal evaporation from OE leaves was 70% of WT after detachment for 10 h (Figure 2e). Leaf tissues in *BnWIN1*-OE were less stained with toluidine blue (TB) than in WT (Figure 3a), suggesting that OE leaves were less damaged than those of WT under salt stress. Moreover, the chlorophyll in salt-stressed *BnWIN1*-OE leaves was leached much slower than in WT leaves; the chlorophyll extracted rate in OE leaves was ~40% of the total amount, whereas that of WT leaves was 68% after incubation in ethanol for 4 h (Figure 3b–d). The results suggest that *BnWIN1*-OE plants had reduced permeability of leaf surface as compared to WT under salt stress. Thus, *BnWIN1*-OE plants display more tolerance to salt stress.

### 2.3. *BnWIN1* Enhanced Wax Accumulation under Salt Stress While It Enhanced Oil Accumulation under Normal Growth Conditions

**Figure 4:**
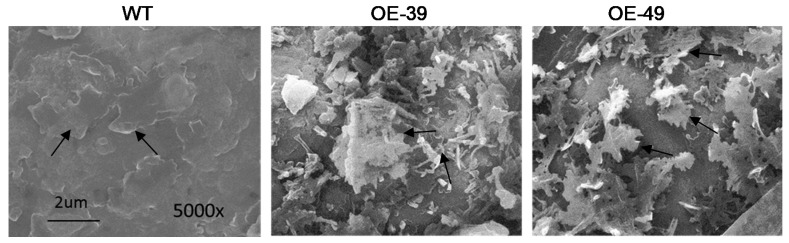
Overexpression of *BnWIN1* enhanced epicuticular wax crystals accumulation under salt stress. Epicuticular wax crystals of leaf surface were observed under scanning electron microscopy. Forty-day-old plants grown in soil in pots were treated with 200 mM NaCl for 30 days and leaf samples were taken for observation. Arrows indicate wax crystals.

**Figure 5:**
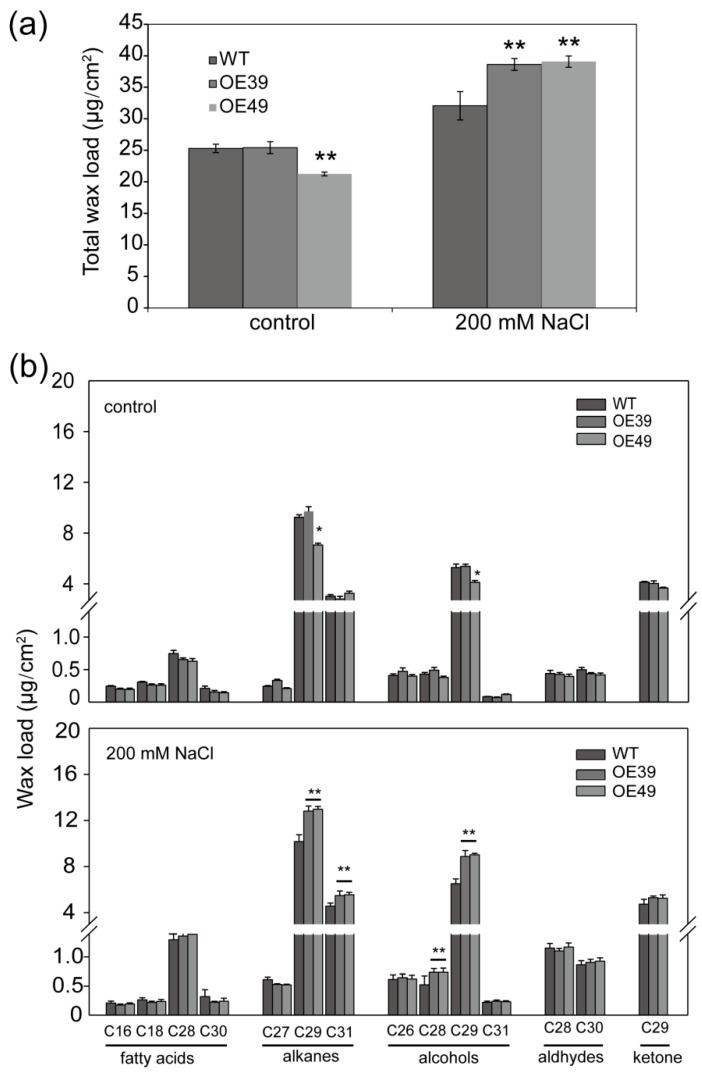
Effect of BnWIN1 on wax content and compositions under salt stress. (**a**) Total wax content in leaves of *BnWIN1*-OE and WT plants under control and salt stress conditions. Values are means ± SD (*n =* 5). (**b**) Wax compositions in leaves of *BnWIN1*-OE and WT plants under control and salt stress conditions. Values are means ± SD (*n =* 5). Forty-day-old plants grown in soil in pots were treated without (control) or with 200 mM NaCl for 30 days. Epicuticular waxes were extracted from leaf surfaces and quantified by GC-MS analysis. * and ** denote significance at *p* < 0.05 and *p* < 0.01, respectively, compared with WT plants based on Student’s *t* test.

To further investigate the effect of *BnWIN1* on wax accumulation, the structure of leaf surface was observed under microscopy. *BnWIN1*-OE leaf surface displayed a heavy deposition of epicuticular wax crystals, exhibiting an enriched typical mixture of platelets, joint-plates, and polygonal rodlets protruding, as compared to WT (Figure 4). To quantify wax accumulation, chloroform extracts from the leaf epicuticular wax were analyzed by GC/MS. Under normal growth conditions, total wax content in *BnWIN1*-OE39 leaf surface was not significantly different from that of WT plants, whereas total wax in *BnWIN1*-OE49 was reduced as compared to WT. When plants were subjected to 200 mM NaCl treatment for 30 days, the total wax content deposited on per unit area of *BnWIN1*-OE leaf surface was significantly higher, with a 20%~22% increase, as compared with WT (Figure 5a). C29-alkane, C31-alkane, C29-alcohol, and C29-ketone are major wax components in *B. napus*. The increased wax accumulation in *BnWIN1*-OE plants resulted from an increase in C29-alkane, C31-alkane, C28-alcohol, and C29-alcohol species. The C29-alkane and C29-alcohol were elevated by 27% and 38%, respectively, relative to WT plants (Figure 5b). The results suggest that BnWIN1 regulates two routes including alkane and alcohol pathways for wax accumulation under salt stress.

**Figure 6:**
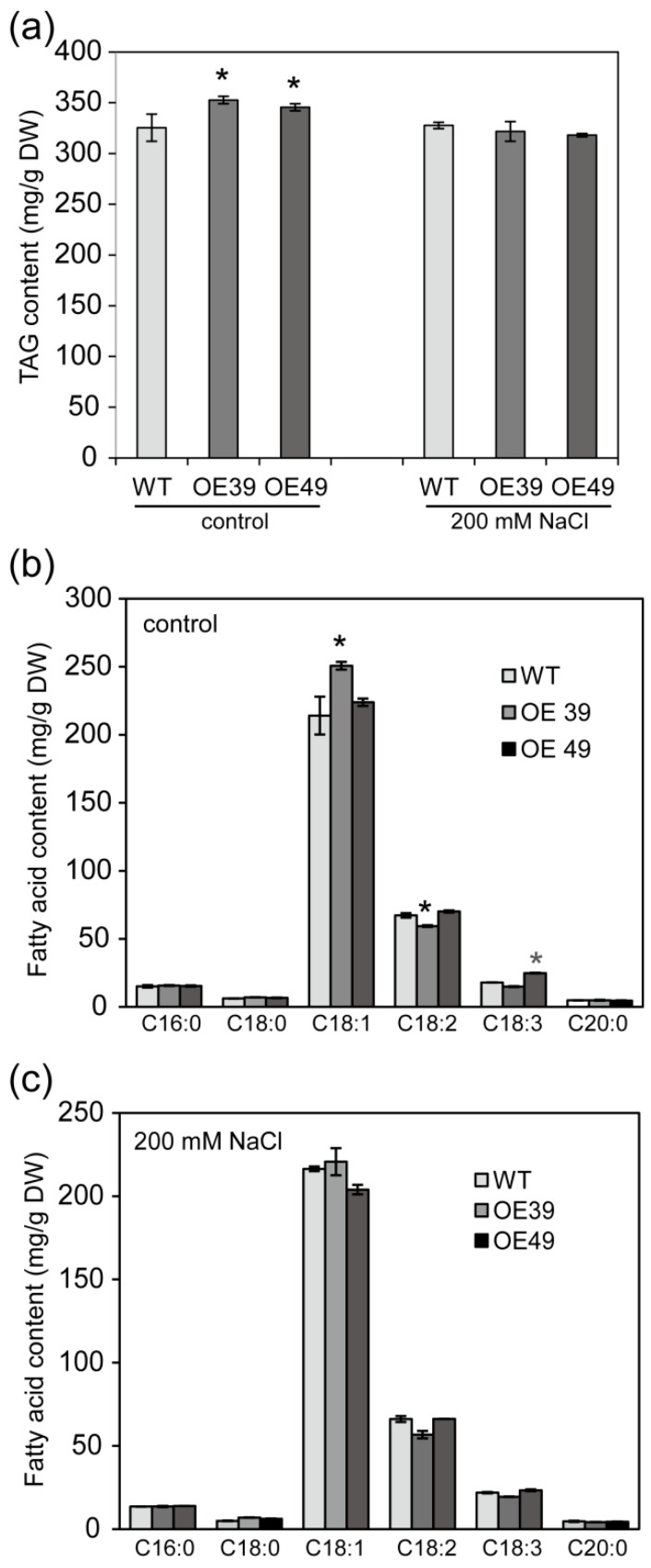
BnWIN1 enhanced seeds oil accumulation. (**a**) Oil (TAG) content of seeds. (**b**,**c**) Fatty acid compositions of seeds. Forty-day-old plants grown in soil in pots were treated without (control) or with 200 mM NaCl for 30 days, and seeds were collected from plants at the mature stage. Seed oil was analyzed by GC after methylation. Values are mean ± SD (*n* = 4 separate samples). * denote significance at *p* < 0.05 compared with WT plants based on Student’s *t* test.

Intracellular and extracellular lipids share fatty acids as common precursors. To test whether enhanced wax accumulation by BnWIN1 led to reduced oil accumulation in seeds, the oil content was measured by GC analysis. Under salt stress, the total oil content of *BnWIN1*-OE seeds was not significantly different from WT seeds (Figure 6a,c). Under normal growth conditions, however, the total oil content of *BnWIN1*-OE seeds was increased by 8% as compared to WT seeds (Figure 6a). The increased oil content in the *BnWIN1*-OE seeds may have resulted from the increase in 18:1 or 18:3 fatty acids (Figure 6b). The results suggest that overexpressing *BnWIN1* leads to enhanced overall lipids, thus, enhanced wax accumulation without a reduction of storage lipids under salt stress and enhanced oil accumulation under normal growth condition.

### 2.4. BnWIN1 Bound to Promoter Regions of Genes Involved in FA, Wax, and Oil Biosynthesis

**Figure 7:**
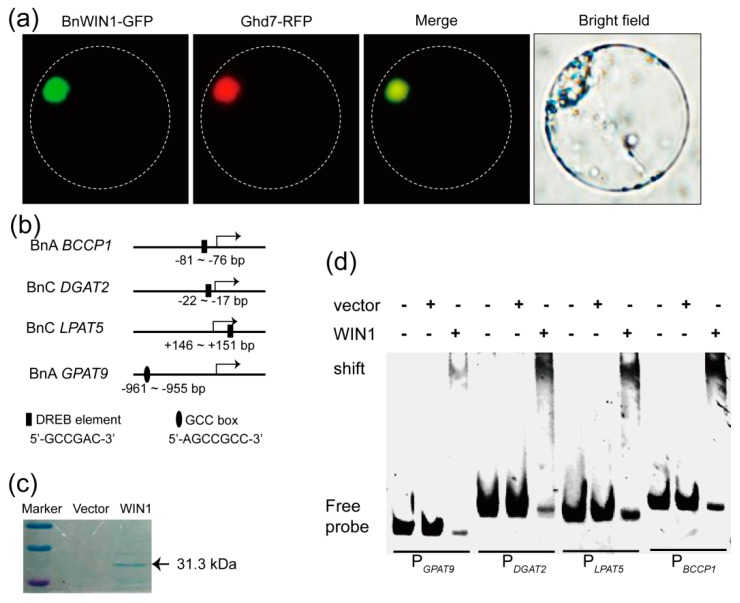
BnWIN1 bound to the promoter region of genes involved in lipid anabolism. (**a**) Nuclear localization of BnWIN1-GFP using BnWIN1-GFP transiently expressed in Arabidopsis protoplasts. Green fluorescence signal of BnWIN1-GFP was overlaid with nuclear red fluorescence (RFP) of the nuclear marker Ghd7-RFP. (**b**) Schematic representation of the cis-elements in promoter regions of target genes. (**c**) Purified recombinant BnWIN1 protein expressed in *E. coli* cells separated by SDS-PAGE gel and stained with Coomassie blue. (**d**) Electrophoretic mobility-shift assay of BnWIN1 binding to the promoter region of *GPAT9*, *DGAT2*, *LPAT5*, and *BCCP1* containing GCC-box or DREB element.

To test the subcellular localization of BnWIN1, the full length *BnWIN1* coding sequence (CDS) was fused with GFP at C-terminus and was transiently expressed in Arabidopsis chloroplasts. The green fluorescent BnWIN1-GFP was visualized under a fluorescence microscope and was overlaid with nucleus marker Ghd7-RFP (Figure 7a), confirming BnWIN1 is localized to the nucleus.

The promoter regions of genes related to lipid anabolic process, such as *BCCP1* in *de novo* FA synthesis [16], *DGAT2* in wax esters [19], *glycerol-3-phosphate acyltransferase 9* (*GPAT9*), and *lysophosphatidic acid acyltransferase 5* (*LPAT5*) in oil synthesis [36,37,38], contain the DREB element (GCCGAC) or GCC box (AGCCGCC) (Figure 7b). To test whether BnWIN1 binds to the target DNA fragments, BnWIN1-His protein was expressed in *E. coli* and purified for binding assays (Figure 7c). The DNA fragments of *P_BCCP1_*, *P_DGAT2_*, *P_LPAT5_*, and *P_GPAT9_*containing the DREB element or GCC box in the promoter regions were amplified for electrophoretic mobility-shift assay (EMSA). When BnWIN1 protein was incubated with the DNA fragment *P_BCCP1_*(187 bp) amplified from the promoter region of *BCCP1,* the target DNA was bound to BnWIN1, as indicated by a shifted band at the top of gel visualized under UV light (Figure 7d). A similar gel shift was observed when BnWIN1 protein was incubated with the DNA fragments, *P_DGAT2_*(180 bp), *P_LPAT5_*(178 bp), and *P_GPAT9_*(156 bp), respectively (Figure 7d). By contrast, the empty vector without BnWIN1 had no such binding (Figure 7d). The results suggest that BnWIN1 binds to the promoter region of genes, including *BCCP1*, *DGAT2*, *LPAT5*, and *GPAT9*.

### 2.5. Overexpression of *BnWIN1* Up-Regulated the Transcript Levels of Genes Related to FA, Wax, and Oil Biosynthesis

**Figure 8:**
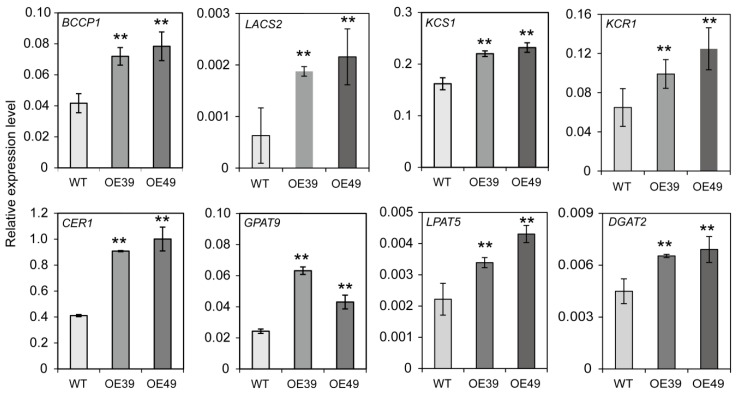
BnWIN1 increased expression of genes related to lipid anabolism. Total RNA was extracted from 40-day-old plants treated with 200 mM NaCl for 12 h. The relative expression levels of genes were analyzed by real-time PCR normalized to *β-Actin* expression level. Values are means ± SD (*n* = 3 separated samples). ** denote significance at *p* < 0.01 compared with WT plants based on Student’s *t* test.

To investigate whether overexpression of *BnWIN1* regulates the transcript level of genes involved in lipid anabolic process, the RNA was extracted from leaves and was analyzed by qRT-PCR. The *BCCP1* transcript was up-regulated and the *BCCP1* expression level in *BnWIN1*-OE plants was more than two-fold compared to WT (Figure 8). The transcript levels of *LACS2, β-ketoacyl-CoA synthase 1* (*KCS1*), *β-ketoacyl-CoA reductase 1* (*KCR1*), and *CER1*, responsible for fatty acid elongation and alkane production, were also substantially higher in *BnWIN1*-OE plants than in WT plants (Figure 8). Overexpressing *BnWIN1* also led to increased expression level of *DGAT2* involved in wax esters. Moreover, the mRNA level of *GPAT9*, encoding a key enzyme in oil synthesis, in *BnWIN1*-OE plants displayed a two-fold increase, as compared to WT (Figure 8). In addition, the expression level of *LPAT5*, a homolog to cotton At-GhLPPAT5 involved in oil synthesis [38], was also up-regulated in *BnWIN1*-OE plants (Figure 8). The results suggest that BnWIN1 synchronously regulates the induction of numerous genes in multiple lipid anabolic blocks including fatty de novo synthesis, fatty acid editing, and lipid assembly for both wax and oil accumulation in *B. napus*.

### 2.6. Overexpression of *BnGPAT9* Enhanced Oil Accumulation in Seeds

**Figure 9:**
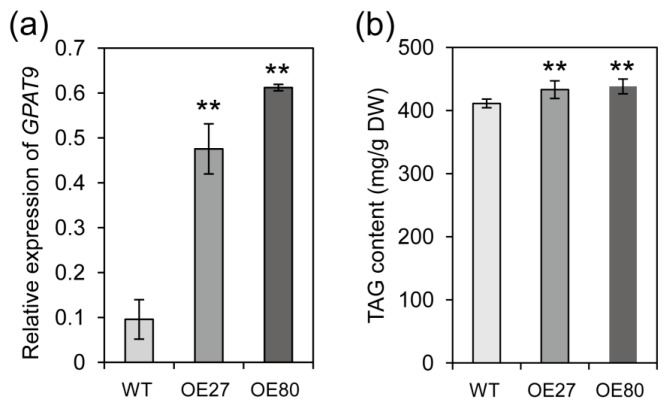
Overexpression of *BnGPAT9* enhanced oil accumulation in seeds. (**a**) The *BnGPAT9* expression level in transgenic plants confirmed by real-time PCR with *β-Actin* as an internal standard. Values are means ± SD (*n* = 3 separated samples). (**b**) Oil content of seeds of *GPAT9*-OE and WT plants. Seeds were collected from plants grown in field at the mature stage. The oil content of seeds was analyzed by GC after methylation. Values are means ± SD (*n* = 4). ** denote significance at *p* < 0.01 compared with WT based on Student’s *t* test.

One novel finding showed that BnWIN1 is also involved in oil accumulation through up-regulating *BnGPAT9* expression. To verify the effect of BnGPAT9 on seed oil synthesis, *BnGPAT9* was cloned and overexpressed in *B. napus* (Figure 9a). The oil content in *BnGPAT9-*OE seeds was significantly higher than in WT seeds (Figure 9b). The results suggest that BnWIN1 up-regulates *BnGPAT9* expression to promote oil accumulation in *B. napus*.

## 3. Discussion

Given the nature of the complicated network of lipid metabolic processes and salt stress response, transcription factors play important roles by simultaneously regulating numerous genes. Here, we show that overexpression of a single gene encoding transcription factor BnWIN1 in *B. napus* could activate multiple genes to enhance wax production and improves plant tolerance to salt stress, whereas it promotes seed oil content under normal growth conditions. These results suggest that BnWIN1 has dual functions with wax and oil synthesis, depending on growth environments. Therefore, increased *BnWIN1* expression in *B. napus* could have significant effects on salt stress tolerance and oil production, which open a potential avenue for the genetic improvement of oil crop plants.

Wax constituents differ in various plant species. VLC *n-*alkanes are predominant components accounting for more than 50% of the wax compounds in Arabidopsis leaves [12], tree tobacco leaves [39], and tomato fruits [40]. Cuticular wax in rice leaves is predominantly comprised of fatty acids, primary alcohols, and aldehydes [9,41]. Wax constituents in Medicago leaves mainly consist of alcohols, and overexpression of Medicago *WXP1*, an AP2 transcription factor, led to an increased C30 primary alcohol [33], indicating that WXP1 regulates the acyl-reduction pathway of wax biosynthesis. Overexpression of Arabidopsis *WIN1*-conferred plants enhanced cuticular wax accumulation with a pronounced increase in alkane components in leaves [7,8], suggesting that Arabidopsis WIN1 is predominantly involved in the decarbonylation pathway [8]. Our results from *B. napus* showed that overexpression of *BnWIN1* led to increased C29-alkane, 31-alkane, C28- alcohol, and C29-alcohol species, as compared to WT. The results suggest that BnWIN1 plays important roles through regulating two routes, the alkane (decarbonylation) and alcohol (the acyl reduction) wax synthesis pathways. In addition, lipid profiling showed that levels of alkanes are much higher than those of fatty acids in wax mixture in *B. napus* leaves. It was suggested that cuticular alkanes play a greater role in water retention than VLCFAs under osmotic stress [42,43].

Fatty acids synthesized in plastids are activated by LACSs to produce acyl-CoAs for further elongation catalyzed by KCS and KCR, generating VLC acyl-CoAs [44,45]. The VLC acyl-CoAs are further reduced to aldehydes and decarbonylated to *n-*alkanes by the CER1/CER3 complex [20,25,26]. Loss of WIN1 led to down-regulation of genes involved in wax accumulation such as KCS1 and CER1 in *Arabidopsis* [7,8,46]. In agreement with the altered wax components, our results show that overexpression of *BnWIN1* up-regulates synergistically the expression levels of numerous genes such as *LACS2*, *KCS1*, *KCR1*, and *CER1*, involved in fatty acid activation, elongation, and decarbonylation for wax accumulation under salt stress. The up-regulation of these wax biosynthesis-related genes can explain the wax accumulation, in particular the increase of VLC *n-*alkanes and alcohols in *BnWIN1*-OE plants under salt stress. Increased wax accumulation restricts surface permeability and water loss, thus enhances plants’ tolerance to osmotic stress.

Extracellular and intracellular lipids share fatty acid precursor pools. We also investigated whether wax accumulation could, directly or indirectly, lead to competition on substrate availability and, ultimately, oil accumulation in seeds. Our results showed that overexpressing *BnWIN1* did not lead to reduced oil content under salt stress. Moreover, unlike what was found in *Arabidopsis* [7,8], overexpression of *BnWIN1* in *B. napu*s plants did not cause growth retardation. The different effects of BnWIN1 and AtWIN1 on allocations to intracellular and epidermal lipids suggest that overexpression *BnWIN1* in *B. napus* may have less competition on fatty acids. WIN1 target genes, such as *CER1*, *KCS1*, and *CER2*, are exclusively related to extracellular lipid synthesis [7,8]. Our study indicates that BnWIN1 regulates directly the induction of genes involved in the synthesis of wax, fatty acids, and seed oils. BnWIN1 contains AP2 domains involved in DNA binding. Our results showed that BnWIN1 was localized to the nucleus, and EMSA data showed that BnWIN1 bound directly to the promoter region of *BnBCCP1*. BCCP is a subunit of ACCase complex that catalyzes rate-limiting reaction to produce malonyl-CoA, which provides a two-carbon donor for *de novo* FA synthesis [14,16] and also for acyl chain elongation to produce VLCFA [14]. Up-regulated *BCCP1* expression in BnWIN1-OE plants promotes de novo fatty acid synthesis by push process and enhances substrate availability for overall lipids accumulation.

It was shown that Arabidopsis WIN1 is responsible for extracellular lipids [7,8]. This study unraveled the novel role of BnWIN1 in the oil accumulation in *B. napus*. Under normal growth condition, overexpresion of *BnWIN1* leads to increased oil content in seeds, which is not found in Arabidopsis WIN1. To separate primary from secondary effects of BnWIN1 on oil accumulation, our results showed that the *BnGPAT9* promoter region possesses a GCC box and *BnGPAT9* expression was up-regulated in *BnWIN1*-OE plants. EMSA data showed that BnWIN1 bound directly to the promoter region of *BnGPAT9*, suggesting that *BnGPAT9* is a direct target of BnWIN1. GPAT9 catalyzes the acylation at the sn-1 position of glycerol-3-phosphate (G3P) to produce LPA and was recently identified to be important for oil accumulation in *Arabidopsis* [36,37]. Our results from *B. napus* plants overexpressing *BnGPAT9* demonstrate that BnGPAT9 plays a positive role in seed oil accumulation. In addition, BnWIN1 also binds to *DGAT2* promoter region. DGAT2 was found to have dual functions for TAG assembly and wax accumulation [19,47,48]. These results suggest that the transcript regulation by BnWIN1 on lipid anabolic process is more comprehensive in *B. napus* as compared to its counterpart in *Arabidopsis*.

In summary, current results showed that BnWIN1 enhanced wax accumulation and salt stress tolerance without apparent side effects on storage lipids and growth. Our data indicate that BnWIN1 regulates the homeostasis between extracellular and intracellular lipids and enhances oil accumulation under normal growth conditions, whereas it enhances wax accumulation to retain water under osmotic stress without at the expense of storage lipids. Our results shed light on the roles of BnWIN1 in lipid regulatory networks and its application in oil crop breeding to improve salt stress tolerance and oil accumulation.

## 4. Materials and Methods

### 4.1. Plant Materials and Growth Conditions

Seeds of canola cultivar (cv.) Westar (*Brassica napus* L.) were sown in plates with moist filter paper or pots with soil. Two-week-old seedlings were transferred to pots containing soil or field under natural conditions during winter/spring seasons in Wuhan, China. For salt treatments, four-week-old seedlings of overexpression and wild type (WT) were transferred to the same pot containing soil and were watered regularly. After recovery for two weeks, the plants were treated with various concentrations (0, 150, 200 mM) of NaCl solution for 30 days in a growth room under 16 h light (25 °C)/8 h dark (20 °C), photosynthetic photon flux density of 200–300 mmol m^−2^ s^−1^, and 60% relative humidity.

### 4.2. RNA Extraction and Quantitative Real-Time PCR

Total RNA was extracted from Westar plant tissues such as roots, leaves, stems, siliques, and developing seeds using Transzol reagent (TransGen Biotech, Beijing, China). RNA extracts were treated with RNase-free DNaseI to remove contaminated DNA. The extracted RNA quality was checked and used as a template for first strand cDNA synthesis by reverse transcriptase using oligo-d (T) 18 primer according the manufacturer’s protocol (TransGen Biotech, Beijing, China). Quantitative real-time PCR was performed as described previously (Hong et al., 2018). Relative expression level was calculated by the comparative Ct method using *Bnβ-Actin* as an internal standard. The primers used are listed in Supplementary Table S1.

### 4.3. Gene Cloning, Vector Construction and Plant Transformation

To produce *BnWIN1*-OE *B. napus* lines, a cDNA pool was synthesized from mRNA extracted from leaves of cv. Westar plants by reverse transcription using a TIANscript RT Kit according to the manufacturer′s instructions (TansGene Biotech, Beijing, China). The full length of *BnWIN1* coding sequence (CDS) was amplified from the cDNA pool by PCR using the primers *BnWIN1* 5′-GGATCCATGGTACAGACGAAGAAGTTC-3′ (forward) and *BnWIN1* 5′-GAGCTCGTTTGTATTGAGAAGCTCCTC-3′ (reverse) and then ligated into the binary vector pBI121 in *BamH* I and *Sac* I cutting site. The resultant construct was transformed into *B. napus* hypocotyls via agrobacterium GV3101 mediation according the method described previously [49]. Transgenic plants were first selected by resistance to kanamycin (50 µg/mL) and were confirmed by PCR using pBI121 vector sequence specific primer 5′-GATGGTTAGAGAGGCTTACGCA-3′ and *BnWIN1* sequence specific primer 5′-GAGCTCGTTTGTATTGAGAAGCTCCTC-3′. *GPAT9*-OE plants were generated via a similar approach. The primers for *GPAT9* CDS cloning are listed in Supplementary Table S1.

### 4.4. Subcellular Localization

The full length of *BnWIN1* CDS was amplified from the cDNA pool of cv. Westar by PCR using primers 5′-GGTACCATGGTACAGACGAAGAAGTTC-3′ (forward) and 5′-GGATCCGTTTGTATTGAGAAGCTCCTC-3′ (reverse) and ligated into pCAMBIA1301s vector at the *Kpn* I and *BamH* I cutting site. The construct containing Bn*WIN1*-GFP expression driven by the 35S promoter was transformed into protoplasts that were isolated from Arabidopsis leaves by polyethylene glycol (PEG)-mediated transient expression system. The construct was also introduced into agrobacterium GV3101, which was filtrated into the leaves of tobacco for 40–50 h for transient protein expression driven by the 35S promoter. The subcellular localization was visualized under a fluorescence microscope (Olympus, BX53, Tokyo, Japan) in Arabidopsis protoplasts for 12 h or tobacco leaves for 40–50 h after infection.

### 4.5. Cuticular Wax Observation by SEM

Leaf piece (1.0 cm^2^) was sampled and fixed with 2.5% glutaraldehyde at 4 °C for 48 h. The samples were dried and coated with palladium–gold in a sputter coater (JEOL JFC-1600, Akishima, Japan). The cuticular wax load was observed under scanning electron microscope (SEM) (QUANTA, Rotterdam, the Netherlands).

### 4.6. Wax Extraction and Analysis

Forty-day-old plants of OE and WT grown in the same pot containing soil were treated with or without 200 mM NaCl for 30 days. Leaf discs with same area (6.872 cm^2^) were sampled from leaves with comparable age and position of OE and WT plants and waxes were extracted from leaf surface with chloroform for 30 s with two repeats. The resultant extracts were dried under a stream of nitrogen and were dissolved in 100 µL of chloroform. Wax constituents containing 10 mg tetracosane internal standard were converted to their trimethylsilyl ethers and esters by adding 20 µL of N, N-bis-trimethylsilyltrifluoroacetamide and 20 µL of pyridine to the extracts and incubating the mixture for 40 min at 70 °C. The fatty acid derivatives were quantitatively analyzed by GC-FID (Agilent Technologies, Santa Clara, CA, USA) and GC-MS (Agilent gas chromatograph coupled to an Agilent 5973 N quadruple mass selective detector) at the Instrumental Analysis Center of Shanghai Jiao Tong University.

### 4.7. Electrophoretic Mobility-Shift Assay

The full length of *BnWIN1* CDS was amplified from the cDNA pool of cv. Westar by PCR using forward primer 5′-GGATCCATGGTACAGACGAAGAAGTTC-3′ paired with reverse primer 5′- CTCGAGGTTTGTATTGAGAAGCTCCTC-3′ and then ligated to pET28a vector after digestion by *BamH* I and *Xho* I. The resultant construct was transformed into *E. coli* strain Rosetta (DE3) and the cells grown in Luria Bertani (LB) medium were induced by 0.6 mM isopropyl-β-D-thiogalactopyranoside (IPTG) to express BnWIN1 at 20 °C. Cells were lysed by sonication in a buffer (300 mM NaCl, 20 mM Tris-HCl, pH 8.0, 10 mM imidazole, 5% glycerol, 50 mM NaH_2_PO_4_) and the cell lysate was centrifuged at 12000 r/min for 20 min. BnWIN1 was purified using Ni-NTA resin according to the manufacturer′s instructions (Sangon, Shanghai, China). Protein from *E. coli* cells containing the pET28a vector only was used as a negative control. Meanwhile, the DNA fragments with approximately 200 bp in the promoter region of related genes such as *BCCP1*, *DGAT2, GPAT9*, and *LPAT5* were amplified from cv. Westar DNA using the primers (Table S1). The amplified target DNA was incubated with purified BnWIN1 in binding buffer (20 mM Tris, pH 8.0, 250 mM NaCl, 2 mM MgCl_2_, 1% glycerol, 1 mg/mL BSA, 1 mM DTT) for 1 h at 4°C. The reaction mixture was separated on native PAGE (6%) and was visualized under UV light.

### 4.8. Seed Oil Measurement

Seeds were dried.in an oven at 60 °C for 3 h to measure dry weight. Seed oil was extracted and methylated with methanol and toluene containing 5% H_2_SO_4_ at 86 °C for 3 h for GC analysis using heptadecanoic acid as an internal standard. The GC running conditions were as follows: the injection port temperature was 180 °C, and the oven temperature was set at 180 °C for 2 min and was increased by 10°C/min up to 220 °C for 5 min. The temperature of the flame ionization detector was 280 °C with flow rates of 30,300, and 25 mL/min for hydrogen, air, and helium, respectively.

## Supplementary Materials

Supplementary materials can be found at https://www.mdpi.com/1422-0067/20/18/4435/s1.

## Abbreviations

*B. napus*	*Brasscia napus*
BCCP	Biotin carboxyl carrier protein
CER	ECERIFERUM
DGAT	Diacylglycerol acyltransferase
EMSA	Electrophoretic mobility shift assay
FAE	Fatty acid elongase
FAS	Fatty acid synthase
EMSA	Electrophoretic mobility shift assay
FAE	Fatty acid elongase
FAS	Fatty acid synthase
GPAT	Glycerol-3-phosphate acyltransferase
KCR	β-Ketoacyl-CoA reductase
KCS	β-Ketoacyl-CoA synthase
LACS	Long chain acyl-CoA synthetase
LPAT	Lysophosphatidic acid acyltransferase
OE	Overexpression
TAG	Triacylglycerol
VLCFA	Very long chain fatty acid
WIN1	WAX INDUCER1/SHINE1
