# Supplementary material for: Overexpression of WAX INDUCER1/SHINE1 Gene Enhances Wax Accumulation under Osmotic Stress and Oil Synthesis in Brassica napus
Source: Int J Mol Sci. 2019 Sep 9;20(18):4435. doi: 10.3390/ijms20184435 (PMC6771042; doi:10.3390/ijms20184435)
Supplement: Supplementary file 1 [file ijms-20-04435-s001.pdf]

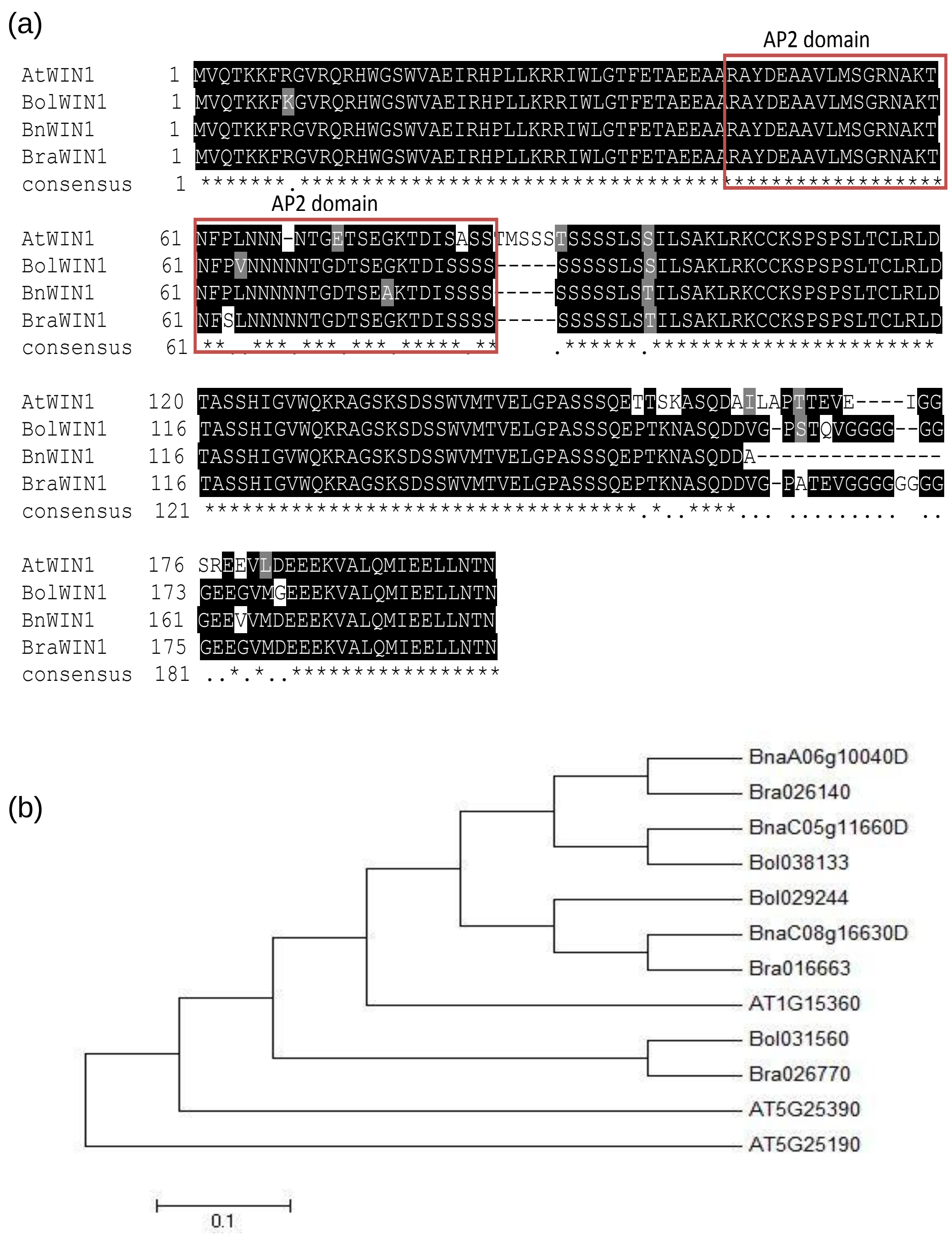

Supplementary Figure 1. Sequence alignment and phylogenetic analysis of WIN1 homologues from different plant species. (**a**) Sequence alignment of WIN1 homologues from *Arabidopsis thaliana* (At), *Brassica oleracea* (Bol), *Brassica napus* (Bn), and *Brassica rapa* (Bra). The red box indicates AP2 domain. (**b**) Phylogenetic analysis of homologous WIN1. The alignment and phylogenetic analysis were conducted using Clustal W and the Neighbor-Joining (NJ) method in MEGA 5 for unrooted phylogeny tree construction. The identical and conserved residues are shaded in black and gray, respectively, by Boxshade program.

Supplementary Table 1. Primers used in this study

**Primer name**	**Primer sequence (5’ → 3’)**	**Purpose**
RT-*Bnactin*-F	TGTTCCCTGGAATTGCTGACCGTA	Real-time PCR
RT-*Bnactin*-R	TGCGACCACCTTGATCTTCATGCT	
BnWIN1-RT-F	CCCGTCAACAACAACAACAAC	
BnWIN1-RT-R	TAGCGCTGAGGATAGAAGAGAG	
BnBCCP1-RT-F	TTCTACTCCTGCCTCTTCACCT	
BnBCCP1-RT-R	TTGCCCCTTCTGCACTTTGT	
BnLPAT5-RT-F	TTCTACTCCAGTGGCCATTTCC	
BnLPAT5-RT-R	GATTGAAATGTGTAGCGGAGGC	
BnDGAT2-RT-F	GCTGGAGAGAGAGTTGAAGGAAG	
BnDGAT2-RT-R	CGAAGACAACACAAGAAGTGGG	
BnCER1-RT-F	GGGTCTCATGAACCAAGGGG	
BnCER1-RT-R	AGCAGAGGCGATGGTGTATG	
BnKCS1-RT-F	GACCGATGGTTCTTCCTTTGTC	
BnKCS1-RT-R	TCGTCGAGAACCGCACTAC	
BnGPAT9-RT-F	TGTTGACGCCTTCTGGAATAG	
BnGPAT9-RT-R	TCCAAGTACCACACTTCACATAC	
BnLACS2-RT-F	TGTTGCTGTCGAAAACCTCG	
BnLACS2-RT-R	CTCTCGGGTACAACCACTGC	
BnKCR1-RT-F	CTCCGACTCGATTCAAGCCA	
BnKCR1-RT-R	TACGAAACCCCAGCGTTGTT	
EMSA-$P_{BnBCCP1}$-F	TTGCCTCTAAAGTATATACAACTGAACATC	Target DNA amplification
EMSA-$P_{BnBCCP1}$-R	AATCTGTTTTCTTGTTGTCGGCTATTT TTC	
EMSA-P_{DGAT2}-F	TGCGTCTGGCAGAGGATTGTCAATGTTTTT	
EMSA-P_{DGAT2}-R	GGGAAGAAGATTTTGAGTCGGTTTGGGTTT	
EMSA-P_{GPAT9}-F	GGTTTGGTCGGGCTTGCCTCTAAGGTTCAC	
EMSA-P_{GPAT9}-R	ATGCGTAAAATCAAGCCGCCGGTAGAGGTC	
EMSA-P_{LPAT5}-F	ATTGTATTCTGATTCTTTCTGTAAGTTTGC	
EMSA-P_{LPAT5}-R	AGAAAAAATGAACAGCCGACCATAATAATC	
GPAT9-OE-F	GGATCCTCTAGAATGAGCAGCACGGCAGGAA	*GPAT9*-OE cloning
GPAT9-OE-F	GAGCTCGGATCCTCACTTGTCTTCCAATCTAGC	
